# Optimizing Metabolite Production Using Periodic Oscillations

**DOI:** 10.1371/journal.pcbi.1003658

**Published:** 2014-06-05

**Authors:** Steven W. Sowa, Michael Baldea, Lydia M. Contreras

**Affiliations:** 1 Microbiology Graduate Program, University of Texas at Austin, Austin, Texas, United States of America; 2 McKetta Department of Chemical Engineering, University of Texas at Austin, Austin, Texas, United States of America; Princeton University, United States of America

## Abstract

Methods for improving microbial strains for metabolite production remain the subject of constant research. Traditionally, metabolic tuning has been mostly limited to knockouts or overexpression of pathway genes and regulators. In this paper, we establish a new method to control metabolism by inducing optimally tuned time-oscillations in the levels of selected clusters of enzymes, as an alternative strategy to increase the production of a desired metabolite. Using an established kinetic model of the central carbon metabolism of *Escherichia coli*, we formulate this concept as a dynamic optimization problem over an extended, but finite time horizon. Total production of a metabolite of interest (in this case, phosphoenolpyruvate, PEP) is established as the objective function and time-varying concentrations of the cellular enzymes are used as decision variables. We observe that by varying, in an optimal fashion, levels of key enzymes in time, PEP production increases significantly compared to the unoptimized system. We demonstrate that oscillations can improve metabolic output in experimentally feasible synthetic circuits.

## Author Summary

Until recently, engineering of microbial strains has been heavily focused on removing or overexpressing individual genes. Although valuable, these efforts do not take into account the potential benefits of tuning enzymes and, thus, do not exploit the full diversity of available synthetic tools to regulate gene expression. Tuning enzyme levels is a key factor in gene expression because optimal levels for an enzyme may change over time in living systems. In this study, we use a kinetic model of *Escherichia coli* to explore how to increase metabolite levels by oscillating sets of enzymes over time. We discover that oscillating relevant clusters of enzymes can increase metabolite levels. When combined with recent experimental studies that demonstrate the ability to build synthetic oscillators and regulators inside living cells, suggesting that oscillating enzymes levels could be of practical relevance to metabolic engineering.

## Introduction

A central goal of synthetic biology is to create new tools and strategies to improve production of metabolites, chemicals and proteins from microbial sources. A particular focus of this field has been the advancement of genetic constructs to exquisitely control gene expression in cells. Traditionally, researchers have modified microbial production strains in a variety of ways that include gene knockouts, gene overexpression, and heterologous pathway expression [1]–[3]. With expanding availability of genome-wide datasets and large metabolic models, emphasis has shifted from single-gene manipulations to genome-wide alterations to improve microbial production processes [4]–[7]. Many of these techniques allow entire clusters of genes to be manipulated simultaneously and are designed to fine tune metabolic regulators for optimal production of a desired product. In this study, we propose a novel method to improve metabolic production of a desired product that relies on time-periodic oscillations of cellular enzymes.

Natural oscillations have been observed in many biological systems [8]–[10]. Early studies have established that oscillations within cellular circuitry can have profound impact on the behavior of a culture [11]. In *E. coli*, oscillations have been studied both experimentally and computationally. Experimentally, glycolytic oscillations have traditionally been generated in response to periodic control of the feed source or external stresses [12]–[16]. Likewise, Chassagnole et al. demonstrated that oscillations observed experimentally could be described using a kinetic model of central carbon metabolism [13], [17]. Additionally, several theoretical studies have shown that oscillations in enzyme levels could increase intracellular concentrations of metabolites in simplified biological circuits [18], [19]. We consider oscillating enzymes within the context of *E. coli* metabolism and suggest ways that these ideas could be implemented experimentally. Computationally, our principal contribution is the use of dynamic optimization to tune these oscillatory responses in a way that maximizes production of a desired metabolite. This contrasts the exclusive use of parameter sensitivity to make control decisions, as used in earlier works [18], [19].

**Figure 1:**
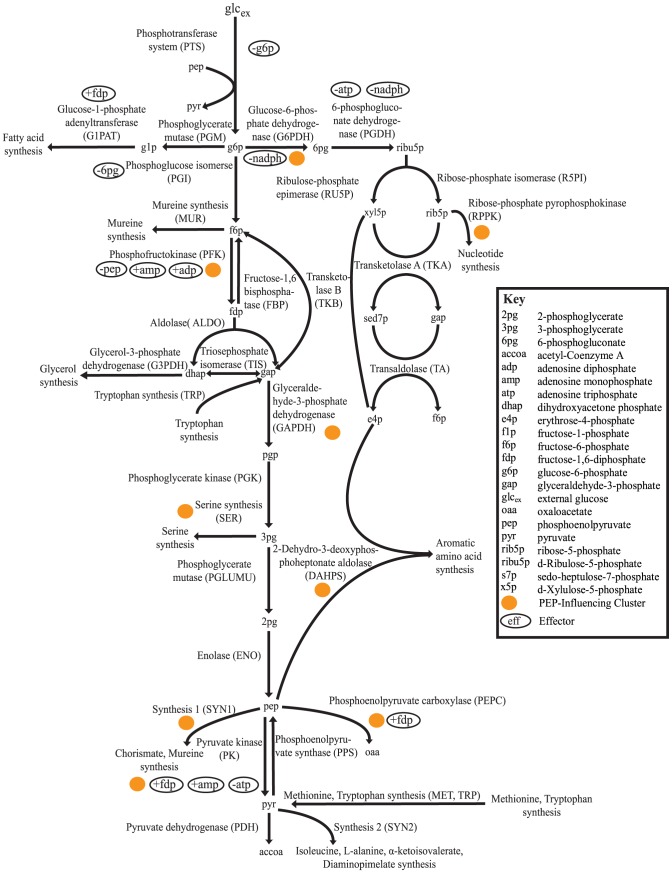
Model system of central carbon metabolism. This kinetic model includes glycolysis, gluconeogenesis, and the pentose phosphate pathway. The model assumes that glucose is the feed substrate and is catabolized into various smaller compounds. Each node is a single metabolite (written in lower case) and these nodes are connected by reactions (written in capital letters). Reactions with multiple metabolites leading into the reaction, such as TKA, TKB, DAHPS, require both substrates for the reaction to occur. Effectors, indicated in white ovals, control the reaction rates of several of the model reactions. Effectors that upregulate a reaction are marked with a +, whereas those that downregulate the reaction are indicated by a -. Enzymes that are most influential for PEP production have been grouped together and called the PEP-influencing cluster (orange circles).

In this work, we explore optimized time-periodic oscillations of a subset of enzymes within a metabolic pathway as a strategy to increase metabolite production. Our hypothesis is based on well-established results from the operation of chemical reactors. This literature shows that the amount of product generated over time can be increased by operating reactors in a non-steady state, time-periodic regime [20], [21] . As a prototype system, we use a modified version of a previously published kinetic model of *E. coli* central carbon metabolism (Figure 1) [17]. Within this system, we explore the use of oscillations in enzyme levels to increase intracellular levels of a key metabolite, phosphoenolpyruvate (PEP). PEP is an important metabolite both in cellular physiology and a key precursor for industrially-important compounds [22]. Specifically, PEP levels control the flow of glucose into the cell and allosterically regulate enzymes within central carbon metabolism [23], [24]. In addition to its cellular functions, PEP is a limiting precursor for microbial production of aromatic amino acids [25], which are important building blocks for products in the chemical, pharmaceutical, and food industries [22], [26]. In light of these facts, many metabolic engineering strategies have been employed to improve PEP availability for aromatic amino acid production [23], [27]–[29]. These studies provide valuable insights into the changes in levels of metabolites, like PEP, that can be achieved through genetic modification.

In this study, we expand an established model of *E. coli* central carbon metabolism [17], [30], [31] by adding two gluconeogenesis reactions from another experimentally-validated model [32] and incorporate enzyme levels into the model using methods described previously [33], [34]. Addition of the phosphoenolpyruvate synthase (PPS) and fructose-1,6-bisphosphatase (FBP) reactions is motivated by previous research results that suggest they are important controllers of PEP levels [25], [35]. Following the incorporation of these reactions, we used sensitivity analysis to identify several key enzymes that impact production of PEP. The levels of these enzymes were then assumed to vary in a periodic fashion, and were modeled as cosine waves whose amplitude, period, and phase properties were optimized to maximize metabolite production (Methods, Optimizations). Finally, we explored the oscillatory properties of an experimentally feasible small synthetic circuit to increase PEP production.

## Results

### Sensitivity analysis identifies key enzymes for PEP production

**Figure 2:**
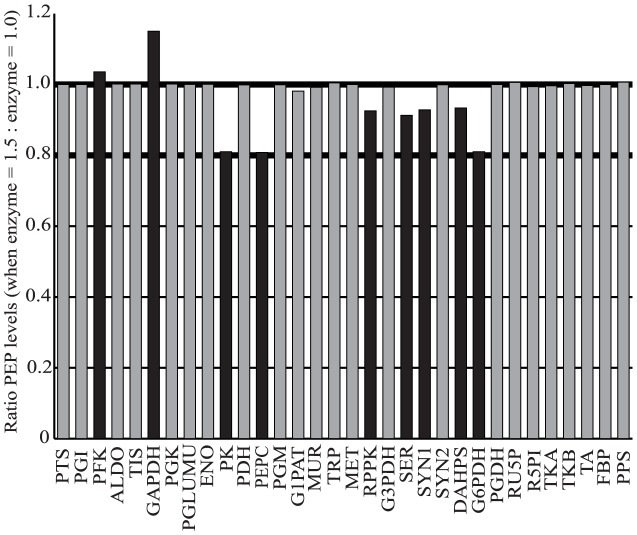
Sensitivity analysis identifies the enzymes most important for PEP production. The bar graph shows the ratio of PEP levels when the indicated enzyme level is raised 50% above its normal, steady state level (a value of 1.5 in the simulation). Upregulated enzymes that caused a change greater than 2% change in PEP levels (black bars) were deemed significant for PEP production.

A key aspect of this study was to identify appropriate enzyme candidates that could be periodically varied in time to increase PEP production. Enzyme levels are incorporated into our model as an additional term to the reaction rate of each enzyme (Methods, Kinetic Model). We assumed that the enzyme levels in the original model were at steady state and arbitrarily set their values to one. As a result, changes in enzyme levels are defined as deviations from this nominal value. To identify enzymes that are important for PEP production, we conducted a sensitivity analysis of the system using step tests. These consisted of monitoring the evolution of PEP levels in time after increasing each enzyme level to be 50% greater than its steady state value (1 to 1.5). Based on this analysis (Figure 2), nine enzymes appeared to affect PEP levels: phosphofructokinase (PFK), glyceraldehyde phosphate dehydrogenase (GAPDH), pyruvate kinase (PK), phosphoenolpyruvate carboxylase (PEPC), ribose-phosphate pyrophosphokinase (RPPK), serine synthesis (SER), synthesis 1 (SYN1), 2-Dehydro-3-deoxyphosphoheptonate aldolase (DAHPS), and glucose-6-phosphate dehydrogenase (G6PDH). Sensitivity analysis indicated that increasing the levels of PFK or GAPDH led to increased PEP levels. This result is somewhat expected, since these two enzymes represent critical branch points for simultaneously controlling flux down the main glycolysis pathway and flux returning from the pentose phosphate pathway.

Individually increasing levels of the remaining seven enzymes resulted in decreased levels of PEP. Four of these enzymes (PEPC, SYN1, DAHPS, and PK) catalyze reactions that directly consume PEP at the PEP node (Figure 1). Two of these enzymes, SER and RPPK, catalyze reactions that are located upstream of the PEP node and direct flux out of the model. Finally, G6PDH is one of the main factors that determines whether the metabolic flux follows glycolysis or the pentose phosphate pathway [17]. The latter three enzymes likely reduce PEP production by playing a more general role in directing metabolic flux out of the boundaries of the system.

### Individual enzyme oscillations result in moderate increases in PEP production

**Figure 3:**
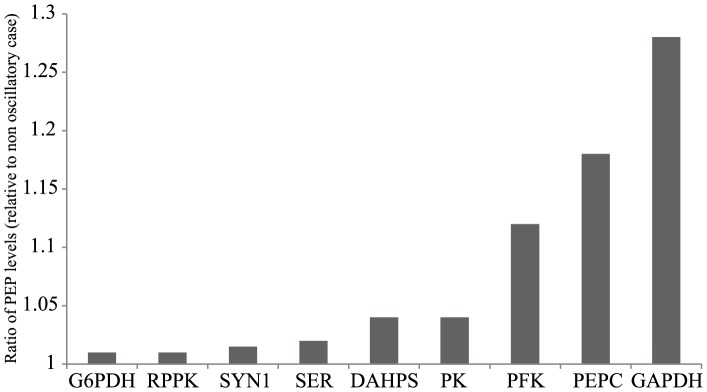
PEP concentration gains by oscillating individual enzymes. A comparison of the increase in total PEP concentration as a result of oscillating each of the indicated enzymes. The enzymes were oscillated by describing their levels as a cosine function and optimizing the properties of each wave. Gain in PEP levels is measured as a ratio of the total PEP concentration in the oscillating case over the total PEP concentration in the time invariant case. All optimizations were run over an 8

Practical implementation of enzyme oscillations (further discussed below) could be achieved through heterologous expression of an enzyme from a plasmid source. In this scenario, it is typical that enzyme concentrations would reach much higher levels than natural expression levels in the cell. To represent the high amount of expression that can be obtained from inducing strong promoters [36], we allowed enzyme levels to reach 20 times their nominal levels in our simulations. With this assumption in place, we first explored independent oscillations of each of the nine PEP-influencing enzymes. As shown in Figure 3, a wide range of increases in total PEP levels (1%–28.3% relative to the case of no oscillation) is observed across the nine enzymes identified as sensitive. While individual enzyme oscillations show smaller improvements than simulated knockouts or over expressions (Table S1), these results showed the potential of oscillating intracellular enzyme levels to positively affect PEP levels compared to the unoptimized case (Figure 3). After examining the benefits of individual oscillations relative to the base case of no oscillation, we reasoned that the oscillating multiple metabolic enzymes simultaneously could further push PEP gains.

### Optimal periodic oscillation of PEP-influencing cluster enzymes further increases PEP levels

We next analyzed the effect of combined oscillations in our system. We grouped all nine sensitive enzymes together into a “PEP-influencing cluster” and focused on optimizing collective expression of this cluster as a unit to mimic natural systems, where large groups of enzymes are co-regulated to produce a specific phenotype [37]. This PEP-influencing cluster represents the theoretical maximum number of enzymes that we hypothesized would significantly influence PEP levels. We modeled oscillations of all the enzymes in the PEP-influencing cluster using simple cosine forcing functions (Methods, Dynamic Optimization). Then, we optimized properties of the waves (i.e. amplitude, frequency and phase) for maximum PEP production. Levels of each enzyme were independently optimized to maximize PEP levels given constraints on metabolite levels derived from experiments (Methods, Dynamic Optimization) [38]–[40] .

**Figure 4:**
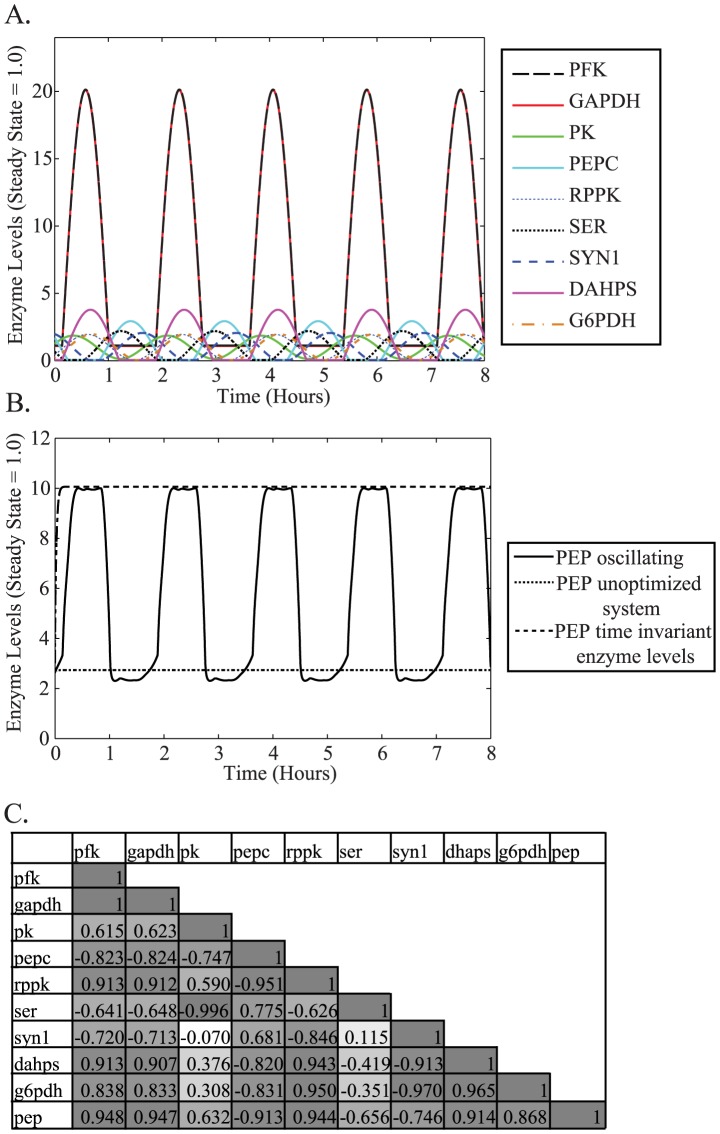
Optimization of PEP-influencing cluster increases intracellular PEP concentration. **A**. Resulting profiles from an optimization of enzyme levels with an eight hour time horizon. PFK (black dashes) overlaps with GAPDH (red line), and all the other enzymes appear distinctly within the plot. Enzyme levels are measured as deviations from steady state (1.0). **B**. Oscillations in PEP levels (black line) resulting from oscillations in enzyme levels. The line with smaller dashes represents the PEP levels without inducing oscillations (unoptimized case). The third line with larger dashes indicates the PEP levels when the levels of enzymes are optimized for PEP production without any oscillations (PEP time invariant enzyme levels). PEP levels are in millimolar. **C**. Correlation coefficients for the oscillating case shown above. The coefficients are colored using a gray scale with the strongest positive (1) and negative (−1) correlations being darkest and weakest correlations colored lightest.

We observed that oscillations of this nine enzyme cluster (Figure 4A), caused PEP levels to oscillate (Figure 4B). These PEP oscillations resulted in a 2.2-fold increase in total PEP levels over an 8 hour time horizon relative to the non-oscillating unoptimized system, suggesting that regulating multiple enzymes in a periodic fashion needed to be further explored as a metabolic optimization strategy. We also compared the oscillatory strategy to a time invariant optimization of the levels of the nine enzymes (Figure 4B, Table S2). The time invariant optimization calculates the PEP gain possible by fine tuning enzyme levels at constant levels. We were encouraged to see that oscillating enzymes produced more PEP than the unoptimized case (Figure 4B). It is important to point out that we constrained PEP levels in our simulations to a 10-fold range (1 mM–10 mM) similar to the total variability of PEP concentrations reported in the literature [40], [41]. We have also verified that describing the variation of enzyme levels in terms of square waves in the optimization calculation leads to a similar increase in the concentration of the PEP (data not shown).

Our optimization revealed that PEP is able to reach higher levels by causing a key enzyme that removes PEP from the system (PEPC) to simultaneously be at low levels while enzymes that help to produce PEP (GAPDH and PFK) are at high intracellular levels. To quantify the relationship between changes in enzyme levels and changes in PEP levels, we calculated correlation coefficients for the time series data corresponding to enzyme levels in the PEP-influencing cluster (Figure 4C). Correlation coefficients indicate how closely changes in one variable (a given enzyme level) correlate to changes in another variable (PEP levels). PFK, GAPDH, PEPC, G6PDH, RPPK and DAHPS form a highly-correlated, synchronized group of enzymes that is primarily responsible for the changes in PEP levels. PK and SER form a secondary enzyme group that also tunes PEP levels (Figure 4C).

### A simple oscillatory circuit can improve metabolic output

Up to this point we had considered the theoretical maximum number of enzymes that could be in our oscillatory circuit to qualitatively evaluate the potential of synthesizing multi-enzyme clusters to improve PEP production. However, given the experimental convenience of manipulating a smaller number of genes, we tested the impact of constructing an smaller oscillatory circuit.

To select the enzymes in this circuit, we analyzed all combinations (of enzymes within the nine enzyme cluster) of two-enzyme clusters to understand which combinations positively impacted PEP the most (data not shown). We did not consider all 32 enzymes for this analysis given the weak influence of most enzymes on PEP levels. We confirmed that individual enzymes that resulted in the largest PEP gains when oscillated independently (i.e. GAPDH, PEPC, or PFK), also produced the largest PEP gains when oscillated with a second enzyme. In particular we noted the largest PEP gain from oscillating GAPDH and PFK simultaneously. To this cluster we added a third enzyme, RPPK (which is essential for cellular viability), to test for additional PEP gains from oscillations that would be difficult to obtain through traditional methods.

**Figure 5:**
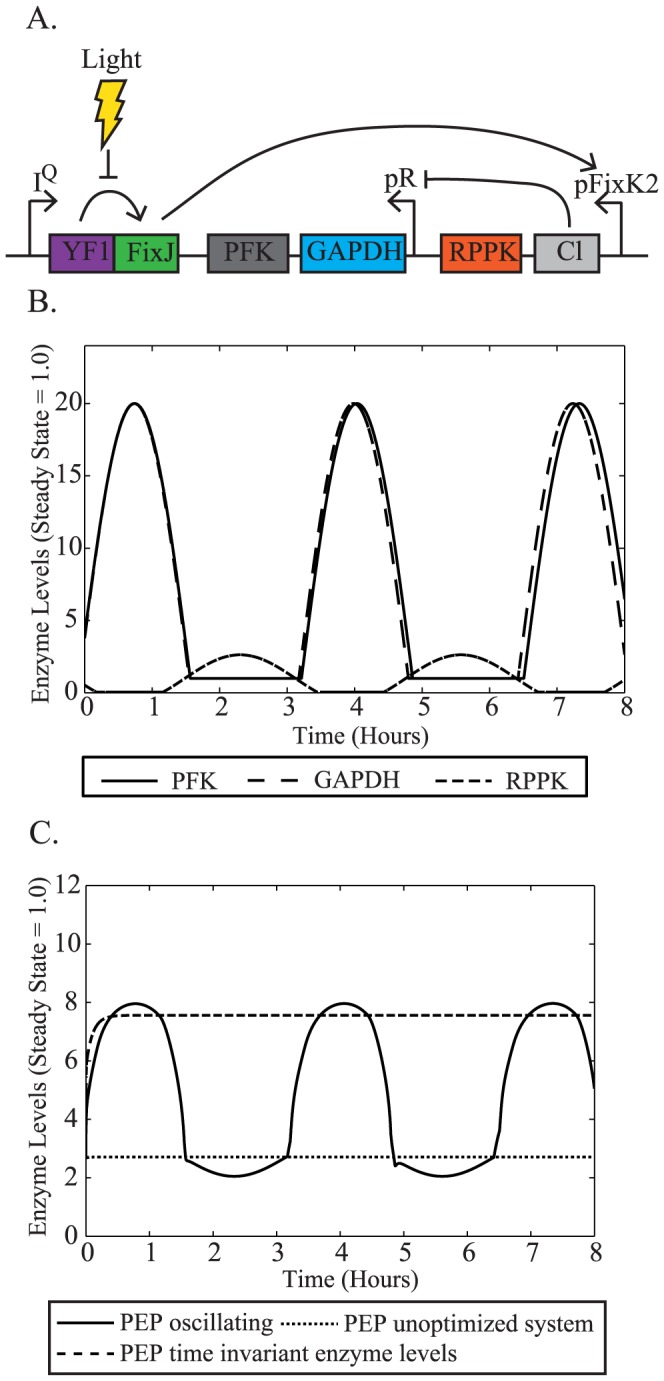
Oscillating the RPPK-GAPDH-PFK cluster resulted in a significant increase in the concentration of PEP. **A**. An illustration of a light-inducible regulatory cluster that could be built for oscillating enzymes. The light-repressed two component system, YF-1/FixJ, expressed constitutively from the promoter I^Q^, controls gene expression from the FixK2 promoter. The lambda repressor protein, Cl, can repress expression from the lambda phage promoter, pR. When the absence of light activates this system, the response regulator, FixJ, is phosphorylated and represses expression of Cl and RPPK, simultaneously increasing expression of GAPDH and PFK. **B**. Resulting enzyme profiles from oscillating RPPK-GAPDH-PFK circuit for 8 hours. Each enzyme was allowed to reach a maximum of 20 times its steady state levels. **C**. PEP levels over 8 hours in response to the oscillating enzymes in B. The line with smaller dashes represents PEP levels without oscillating any enzymes (unoptimized case). The line with larger dashes shows the PEP from optimizing the levels of the three enzymes rather than oscillating (PEP time invariant enzyme levels).

We optimized periodic expression of RPPK, GAPDH, and PFK by simulating these genes in a recently described light-inducible system [42] (Figure 5A). In this circuit, the bacterial two-component system, YF1 (histidine kinase repressed by light)/FixJ (response regulator), represses the expression of transcripts from the FixK2 promoter. A second repressor protein, lambda phage cl, is expressed from the FixK2 promoter which represses the lambda phage promoter pR. A reversible physical input (i.e. light) simultaneously represses production of genes controlled by FixK2, and cause pR promoter genes to be expressed since the promoter is no longer being repressed. Importantly, in the presence of light, GAPDH and PFK genes are expressed and RPPK is suppressed simultaneously.

The optimized circuit (Figure 5B) showed a significant increase in PEP levels (1.86-fold increase in total PEP concentration) relative to the levels observed in the unoptimized case with no oscillations (Figure 5C). Although oscillating the cluster produced less PEP than time-invariant optimization of the three enzyme levels, we were encouraged by the fact that the oscillating three-enzyme circuit produced far more PEP than the unoptimized case and 85% as much PEP as the oscillating nine-enzyme circuit (Figure 5C, Table S3). This data validated the potential of selecting influential enzyme clusters and of periodically oscillating them as a way to increase targeted production of a metabolite of interest.

## Discussion

We have shown that tuned periodic oscillations of selected enzyme levels in a metabolic pathway can have a positive effect on metabolite production. These findings agree with observations made in chemical reactors that a higher cumulative yield of product can be reached by operating the reactor in a periodic fashion [20], [21], [43]. In this study, we report an experimentally viable three enzyme oscillating cluster that can lead to a 1.86-fold increase in PEP production compared to the original (unoptimized) system.

The motivation of this work was to evaluate the tradeoff between drastic alterations in gene expression and more moderate metabolic changes (i.e. oscillations) that can lessen metabolic burden and tune essential genes. A key question was how the levels of PEP increase obtained by periodic enzyme oscillations compare to traditional strategies of genetic deletions and enzyme overexpression. We expected that constitutive overexpression of enzymes over time (where levels are always at a maximum) would lead to higher PEP levels than the periodic oscillation cases (where those maximum enzyme levels are only periodically achieved). On average an increase of 32% in PEP levels was observed by deleting individual genes that were negatively correlated with PEP production, relative to the oscillation of these same individual genes. A similar trend was discovered when individual enzymes that positively correlated with PEP production were constitutively overexpressed relative to when they were independently oscillated.

To gauge the accuracy of our projections we compared the results of our simulated knockouts and overexpression to experimental data on these modifications [2], [29], [44]–[46]. This comparison shows that, while qualitatively correct, our model is significantly underestimating the metabolite concentration increases gained by mutant strains (i.e. our model projects a PEPC (*ppc*) mutant to have a 1.67-fold increase in PEP concentration, but experimental data shows that the knockout produces a 3-fold increase [40]). We suspect that, likewise, our model underestimates the gains in PEP levels obtained from oscillatory simulations.

We believe that oscillatory strategies could prove valuable for several reasons. First, oscillations provide an additional way to manipulate expression of essential genes. Second, this approach can reduce the metabolic burden in cells that is observed as a result of constitutive overexpression of multiple proteins. Although our model cannot capture these effects, it has been well established that consistently overexpressed proteins can become a large metabolic burden on the cell representing as much as 15–40% of the total cellular protein produced by recombinant cells [47]–[49]. Third, oscillations could be valuable in situations where there are growth tradeoffs in producing the final product. For example, if the final product is toxic to cell growth [50] or causes cells to form spores [51], then enzyme oscillations could allow cells some recovery time and prolong their viability. Finally, oscillatory strategies could also be valuable if recombinant enzymes are oscillated synchronously with natural periodic rhythms found in many cells types [52]–[54]. We also envision using oscillatory strategies to tune global regulatory genes resulting in the simultaneous coordination of many genes and pathways. For these reasons, a dynamic strategy can provide a complementary approach to current methods depending on the particular metabolic optimization problem. The method outlined here is generally applicable to any organism that is genetically pliable and for which a kinetic model can be constructed.

Oscillating enzyme levels inside cells requires (1) a method to induce periodic changes *in vivo* and (2) the ability to create and manipulate regulatory clusters. Since the creation of the Goodwin oscillator [55] in the 1960s, researchers have been creating more robust and sophisticated synthetic oscillators [56]. The Repressilator, is another created by Elowitz and Leibler, is a good example of a synthetic oscillator, where each of the three genes inhibit transcription of its successor and cause sustained oscillations to form [57]. These simple oscillators have led to the development of a fast, robust tunable synthetic oscillator inside living cells [56]. In addition to these oscillators, light inducible systems have shown the potential to modulate gene expression in a highly controllable fashion [58], [59]. While many synthetic oscillatory systems have been rigorously tested computationally and validated experimentally, their incorporation into larger regulatory circuitry has been less explored. The number of innovations in the engineering of synthetic circuits to control gene expression *in vivo* is expected to continue to rise.

Manipulation of regulatory clusters, whether naturally occurring or rationally designed, has already proven to be an effective method to improve metabolite production [4], [27]. Changes in system and flux profiles can be achieved by altering global regulatory systems, including methods such as knocking out transcriptional regulators [60], tuning promoters [5], and altering post transcriptional regulatory systems [4]. Some of these methods have already been applied to increase carbon flow through the PEP node. For instance, by manipulating the Carbon Storage Regulator system using overexpression and knockouts, intracellular PEP levels can be increased 2 to 3-fold [27], [61]. Oscillating components of regulatory circuits, like parts of the Carbon Storage Regulator system, provides the potential additional advantage of bypassing the negative impact of multiple gene deletions and/or gene overexpression on cell growth that has been widely reported in the literature.

Oscillating enzymes levels could be a useful strategy for improving production of metabolites in conjunction with traditional methods. Oscillations would be ideal when controlling the levels of genes that hinder or completely impair cellular growth, including many genes in central carbon metabolism [62]. These oscillatory clusters can tune and coordinate expression of multiple cellular enzymes. It is important to note that oscillations of individual proteins could be further customized by tuning the promoters, altering ribosome binding sites, and using different protein degradation tags to change the rate of degradation for each protein. Exploiting customized regulatory “parts” to creatively control gene expression has become common in the construction of synthetic genetic circuits [63].While initial synthetic oscillatory circuits may take the simpler form similar to the RPPK-GAPDH-PFK circuit, our future research will also consider larger circuits that tune multiple genes directly from the chromosome.

## Methods

### Kinetic model

The kinetic model used in this study is an adaptation of the model created by Chassagnole et al. [17] (Figure 1). The metabolite concentrations are modeled by dynamic mass balance equations, resulting in a set of ordinary differential equations of the form: 
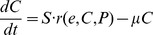
(1)where S is the *n* x *m* dimensional stoichiometric matrix, r is an m-dimensional vector of reaction rates, C is an n-dimensional vector of metabolite concentrations, P is a k-dimensional parameter vector, and *e* is an m-dimensional vector of enzyme levels. The second term is a dilution factor that accounts for biomass generation demands on that metabolite (μ = specific growth rate).

One of the drawbacks of the original model [17] is that the accumulation of co-metabolites (ATP, NAD, etc.) is expressed as explicit time dependent functions rather than as mass balance equations. Since we ran simulations over a significantly longer time frame than originally modeled, we removed the time-dependent descriptions of the co-metabolites and replaced them with constant values following the approach taken in other studies [32], [34].

Enzyme levels were added to the originally published model [17] by introducing an additional term, 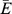, into the reaction rate (r_enz_) equations: 
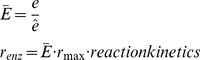
(2)Where r_max_ is the maximum reaction rate, ê is the steady state enzyme concentration (arbitrarily set to equal one) and e is the current enzyme concentration, such that enzyme levels (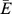) are observed as deviations from their steady state values similar to previous studies [33], [34].

### Incorporating gluconeogenic reactions

The model contains mass balance equations for 18 metabolites in the glycolysis, gluconeogenesis, and pentose phosphate pathways. It contains 32 reactions, including two gluconeogenic reactions catalyzed by phosphoenolpyruvate synthase (PPS) and fructose-1,6-bisphosphatase (FBP), which were taken from a previous study [32]. The parameters for these equations were taken directly from the aforementioned work of Usuda et al.[32] with the exception of the r_max_ values which were recalculated using the top-down approach previously described by Rizzi et al. [64]. A table of all parameters, equations and modified mass balance equations is provided in the supplementary materials (Text S1).

### Optimizations

A sensitivity analysis of enzyme levels on PEP levels was carried out by performing a battery of step tests on the dynamic model. Specifically, with the model initially at its nominal steady state, the concentration of each enzyme was increased to 1.5 times its steady state value; the model was subsequently simulated for a sufficiently long period of time for a new steady state to be reached. The subset of enzymes that led to more than a 2% change in PEP levels became the targets of the optimization. For the optimization calculations, it was assumed that the levels of each key enzyme vary in time following a simple sinusoidal function,
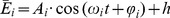
(3)Where *A* is the amplitude, *t* is time, *ω* is the frequency, *φ* is the phase, and *h* is a constant bias (*h* = 1).

gPROMs [65] was used to determine the optimal values of these parameters. The optimization was formulated as:
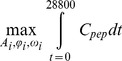
Subject to:

Process model equations 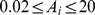


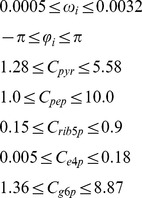
(4)Where C_metabolite_ represents the concentration of metabolites in millimoles/liter, ω in radians/second, φ in radians.

Constraints on the amplitudes of the enzyme level waves allow enzymes to have a maximum of 20 times their steady state concentration (Lower bound: 1/20^th^). We assumed that enzymes that improve PEP production (GAPDH and PFK) would be added to the oscillatory circuit on a plasmid without modifying the genomic copies of the gene. To model this assumption, the levels of GAPDH and PFK were not allowed to pass below their original steady state levels of 1.0. The constraints were within the fold changes in enzyme levels described in experimental studies [39], [66].

Constraints for metabolite concentrations (i.e. *C_pyr_*, *C_pep_*, *C_rib5p_*, *C_e4p_*, and *C_g6p_*) were set using the fold change observed in experimental studies as constraints [38], [40]. These studies were chosen because they consider large scale *E. coli* responses to perturbations and metabolic profile changes that could be achieved by manipulating artificial regulatory systems.

As a control to compare the performance of the dynamic optimizations, time invariant optimizations were also run by replacing the time dependent enzyme level descriptions (Equation 3) with a constant term for the enzyme level (h). These constant levels were then used as the optimization variables to maximize PEP production. In these optimizations, all enzymes were allowed to vary between 0 and 20 except for essential enzymes which were allowed to vary between 0.25 and 20. With these exceptions all other constraints and conditions controlling the dynamic optimization were applied to the static optimizations.

### Correlation coefficients

Correlation coefficients for the time series corresponding to enzyme and metabolite levels were calculated in MATLAB based on the Pearson product moment correlation coefficient formula:
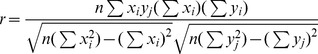
(5)Where *x* is a matrix of the time-dependent enzyme levels of enzyme *i* and *y* is a matrix of the time-dependent enzyme levels of enzyme *j*. The resulting matrix was then colored using a grayscale to indicate the highest and lowest correlation values. A correlation coefficient close to one indicates a strong correlation.

## Supporting Information

Table S1 **Table containing increases in PEP levels obtained by knocking out or overexpressing individual genes.** The increase in PEP levels is measured as the ratio of the total PEP produced in the simulated knockout or overexpression over 8 hours divided by the total PEP in the unoptimized system. Individual gene knockouts were simulated by setting the corresponding enzyme level to zero for the course of the simulation. Knockouts of essential genes were not simulated. Over expressions were simulated by setting the enzyme level to 100 times its nominal value for 8 hours.
(XLSX)

Table S2 **Table containing the optimal enzyme levels for the nine enzyme circuit computed assuming that the levels are constant in time.** Enzymes were allowed to vary between the levels of 0 to 20 (except for essential enzymes which varied between 0.25 to 20) in order to maximize PEP production. The PEP levels over the course of this optimization are shown as a long-dashed line in Figure 4B.
(XLSX)

Table S3 **Table containing the optimal enzyme levels for the three enzyme circuit assuming the levels are constant in time.** A steady state optimization was used to maximize PEP production by varying the levels of each enzyme between 0 and 20 (except for essential enzymes which varied between 0.25 and 20). The PEP produced from this optimization in indicated by a long-dashed line in Figure 5C.
(XLSX)

Text S1 **Docx file of parameters, equations and mass balance equations used in this work.**
(DOCX)
