# Supplementary material for: Optimizing Metabolite Production Using Periodic Oscillations
Source: PLoS Comput Biol. 2014 Jun 5;10(6):e1003658. doi: 10.1371/journal.pcbi.1003658 (PMC4046915; doi:10.1371/journal.pcbi.1003658)
Supplement: Text S1 — Docx file of parameters, equations and mass balance equations used in this work. (DOCX) [file pcbi.1003658.s004.docx]

**Rate Equations**

__

__

__

$$A=1+\frac{C_{pep}}{K_{PFK,pep}}+\frac{C_{adp}}{K_{PFK,adp,b}}+\frac{C_{amp}}{K_{PFK,amp,b}}$$

$$B=1+\frac{C_{adp}}{K_{PFK,adp,b}}+\frac{C_{amp}}{K_{PFK,amp,a}}$$

$$r_{Mur}=ENZ_{Mur}\cdot r_{Mur}^{\max}$$

$$r_{Trp}=\bar{E}_{Trp}\cdot r_{Trp}^{\max}$$

$$r_{Met}=\bar{E}_{Met}\cdot r_{Met}^{\max}$$

$$r_{Ru5p}=\bar{E}_{Ru5p}\cdot r_{Ru5p}^{\max}\left(C_{ribu5p}-\frac{C_{xyl5p}}{K_{Ru5P,eq}}\right)$$

$$r_{R5P1}=\bar{E}_{R5P1}\cdot r_{R5P1}^{\max}\left(C_{ribu5p}-\frac{C_{rib5p}}{K_{R5P1,eq}}\right)$$

$$r_{TKa}=\bar{E}_{TKa}\cdot r_{TKa}^{\max}\left(C_{rib5p}C_{xyl15p}-\frac{C_{sed7p}C_{gap}}{K_{TKa,eq}}\right)$$

$$r_{TKb}=\bar{E}_{TKb}\cdot r_{TKb}^{\max}\left(C_{xyl15p}C_{e4p}-\frac{C_{f6p}C_{gap}}{K_{TKb,eq}}\right)$$

$$r_{TA}=\bar{E}_{TA}\cdot r_{TA}^{\max}\left(C_{gap}C_{sed7p}-\frac{C_{e4p}C_{f6p}}{K_{TA,eq}}\right)$$

**Periodic Forcing Function**

$$\bar{E}_{i}=A_{i}\cdot\cos(\omega_{i}+\phi_{i})+h$$

**Mass Balance Equations**

$$\frac{dC_{glc}^{extracellular}}{dt}=D\left(C_{glc}^{feed}-C_{glc}^{extracellular}\right)+f_{pulse}-\frac{C_{x}r_{PTS}}{\rho_{x}}$$

$$\frac{dC_{g6p}}{dt}=65\cdot r_{PTS}-r_{PGI}-r_{G6PDH}-r_{PGM}-\mu C_{g6p}$$

$$\frac{df6p}{dt}=r_{PGI}-r_{PFK}-r_{TKB}-r_{TA}-r_{MUR}+r_{FBP}-\mu C_{f6p}$$

$$\frac{dfdp}{dt}=r_{PFK}-r_{ALDO}-r_{FBP}-\mu C_{fdp}$$

$$\frac{dC_{gap}}{dt}=r_{ALDO}+r_{TIS}-r_{GAPDH}+r_{TKa}+r_{TKb}-r_{TA}+r_{Trp}-\mu C_{gap}$$

$$\frac{dC_{dhap}}{dt}=r_{ALDO}-r_{TIS}-r_{G3PDH}-\mu C_{dhap}$$

$$\frac{dC_{pgp}}{dt}=r_{GAPDH}-r_{PGK}-\mu C_{pgp}$$

$$\frac{dC_{3pg}}{dt}=r_{PGK}-r_{PGluMu}-r_{Ser}-\mu C_{3pg}$$

$$\frac{dC_{2pg}}{dt}=r_{PGluMu}-r_{ENO}-\mu C_{2pg}$$

$$\frac{dpyr}{dt}=r_{PK}+65\cdot r_{PTS}-r_{PDH}-r_{SYN2}-r_{MET}+r_{TRP}-r_{PPS}-\mu C_{pyr}$$

$$\frac{dpep}{dt}=r_{ENO}-65\cdot r_{PTS}-r_{PK}-r_{PEPC}-r_{DAHPS}-r_{SYN1}+r_{PPS}-\mu C_{pep}$$

$$\frac{dC_{6pg}}{dt}=r_{G6PDH}-r_{PGDH}-\mu C_{6pg}$$

$$\frac{dC_{ribu5p}}{dt}=r_{PGDH}-r_{Ru5P}-r_{R5P1}-\mu C_{ribu5p}$$

$$\frac{dC_{xy15p}}{dt}=r_{Ru5P}-r_{TKa}-r_{TKb}-\mu C_{xy15p}$$

$$\frac{dC_{sed7p}}{dt}=r_{TKa}-r_{TA}-\mu C_{sed7p}$$

$$\frac{dC_{rib5p}}{dt}=r_{R5P1}-r_{TKa}-r_{RPPK}-\mu C_{rib5p}$$

$$\frac{dC_{e4p}}{dt}=r_{TA}-r_{TKb}-r_{DAHPS}-\mu C_{e4p}$$

$$\frac{dC_{glp}}{dt}=r_{PGM}-r_{GIPAT}-\mu C_{glp}$$

**Equation Parameters**

| Equation | Parameter | Value | Referemce |
| --- | --- | --- | --- |
| PTS | k_pts,a1_ | 3082.3 mM | Chassagnole, et al. (2002). |
|  | k_pts,a2_ | 0.01 mM | Chassagnole, et al. (2002). |
|  | k_pts,a3_ | 245.3 | Chassagnole, et al. (2002). |
|  | k_pts,g6p_ | 2.15 mM | Chassagnole, et al. (2002). |
|  | n_pts,g6p_ | 3.66 | Chassagnole, et al. (2002). |
|  | r_max,pts_ | 7829.78 | Chassagnole, et al. (2002). |
| PGI | k_pgi,g6p_ | 2.90 mM | Chassagnole, et al. (2002). |
|  | k_pgi,f6p_ | 0.266 mM | Chassagnole, et al. (2002). |
|  | k_pgi,eq_ | 0.1725 | Chassagnole, et al. (2002). |
|  | k_pgi,g6p,6pginh_ | 0.2 mM | Chassagnole, et al. (2002). |
|  | k_pgi,f6p,6pginh_ | 0.2 mM | Chassagnole, et al. (2002). |
|  | r_max,pgi_ | 650.98 | Chassagnole, et al. (2002). |
| PFK | k_pfk,f6p,s_ | 0.325 mM | Chassagnole, et al. (2002). |
|  | k_pfk,atp,s_ | 0.123 mM | Chassagnole, et al. (2002). |
|  | k_pfk,adp,a_ | 128.0 mM | Chassagnole, et al. (2002). |
|  | k_pfk,adp,b_ | 3.89 mM | Chassagnole, et al. (2002). |
|  | k_pfk,adp,c_ | 4.14 mM | Chassagnole, et al. (2002). |
|  | k_pfk,amp,a_ | 19.1 mM | Chassagnole, et al. (2002). |
|  | k_pfk,amp,b_ | 3.20 mM | Chassagnole, et al. (2002). |
|  | k_pfk,pep_ | 3.26 mM | Chassagnole, et al. (2002). |
|  | l_pfk_ | 5629067 | Chassagnole, et al. (2002). |
|  | n_pfk_ | 11.1 | Chassagnole, et al. (2002). |
|  | r_max,pfk_ | 1840.58 | Chassagnole, et al. (2002). |
| ALDO | k_aldo,fdp_ | 1.75 mM | Chassagnole, et al. (2002). |
|  | k_aldo,dhap_ | 0.088 mM | Chassagnole, et al. (2002). |
|  | k_aldo,gap_ | 0.088 mM | Chassagnole, et al. (2002). |
|  | k_aldo,gap,inh_ | 0.6 mM | Chassagnole, et al. (2002). |
|  | v_aldo,blf_ | 2 | Chassagnole, et al. (2002). |
|  | k_aldo,eq_ | 0.144 mM | Chassagnole, et al. (2002). |
|  | r_max,aldo_ | 17.41 | Chassagnole, et al. (2002). |
| TIS | k_tis,dhap_ | 2.8 mM | Chassagnole, et al. (2002). |
|  | k_tis,gap_ | 0.3 mM | Chassagnole, et al. (2002). |
|  | k_tis,eq_ | 1.39 | Chassagnole, et al. (2002). |
|  | r_max,tis_ | 68.67 | Chassagnole, et al. (2002). |
| GAPDH | k_gapdh,gap_ | 0.683 mM | Chassagnole, et al. (2002). |
|  | k_gapdh,pgp_ | 0.0000104 mM | Chassagnole, et al. (2002). |
|  | k_gapdh,nad_ | 0.252 mM | Chassagnole, et al. (2002). |
|  | k_gapdh,nadh_ | 1.09 mM | Chassagnole, et al. (2002). |
|  | k_gapdh,eq_ | 0.63 | Chassagnole, et al. (2002). |
|  | r_max,gapdh_ | 921.59 | Chassagnole, et al. (2002). |
| PGK | k_pgk,pgp_ | 0.0468 mM | Chassagnole, et al. (2002). |
|  | k_pgk,3pg_ | 0.473 mM | Chassagnole, et al. (2002). |
|  | k_pgk,adp_ | 0.185 mM | Chassagnole, et al. (2002). |
|  | k_pgk,atp_ | 0.653 mM | Chassagnole, et al. (2002). |
|  | k_pgk,eq_ | 1934.4 | Chassagnole, et al. (2002). |
|  | r_max,pgk_ | 3021.8 | Chassagnole, et al. (2002). |
| PGLUMU | k_pglumu,3pg_ | 0.2 mM | Chassagnole, et al. (2002). |
|  | k_pglumu,2pg_ | 0.369 mM | Chassagnole, et al. (2002). |
|  | k_pglumu,eq_ | 0.188 | Chassagnole, et al. (2002). |
|  | r_max,pglumu_ | 89.04 | Chassagnole, et al. (2002). |
| ENO | k_eno,2pg_ | 0.1 mM | Chassagnole, et al. (2002). |
|  | k_eno,pep_ | 0.135 mM | Chassagnole, et al. (2002). |
|  | k_eno,eq_ | 6.73 mM | Chassagnole, et al. (2002). |
|  | r_max,eno_ | 330.44 | Chassagnole, et al. (2002). |
| PK | k_pk,pep_ | 0.31 mM | Chassagnole, et al. (2002). |
|  | k_pk,adp_ | 0.26 mM | Chassagnole, et al. (2002). |
|  | k_pk,atp_ | 22.5 mM | Chassagnole, et al. (2002). |
|  | k_pk,fdp_ | 0.19 mM | Chassagnole, et al. (2002). |
|  | k_pk,amp_ | 0.2 mM | Chassagnole, et al. (2002). |
|  | l_pk_ | 1000 | Chassagnole, et al. (2002). |
|  | n_pk_ | 4 | Chassagnole, et al. (2002). |
|  | r_max,pk_ | 0.061 | Chassagnole, et al. (2002). |
| PDH | k_pdh,pyr_ | 1159 mM | Chassagnole, et al. (2002). |
|  | n_pdh_ | 3.68 mM | Chassagnole, et al. (2002). |
|  | r_max,pdh_ | 6.059 | Chassagnole, et al. (2002). |
| PEPCXYLASE | k_pep,cxylase,pep_ | 4.07 mM | Chassagnole, et al. (2002). |
|  | k_pep,cxylase,fdp_ | 0.700 mM | Chassagnole, et al. (2002). |
|  | n_pep,cxylase,fdp_ | 4.21 | Chassagnole, et al. (2002). |
|  | r_max,pepcxylase_ | 0.107 | Chassagnole, et al. (2002). |
| PGM | k_pgm,g6p_ | 1.038 mM | Chassagnole, et al. (2002). |
|  | k_pgm,g1p_ | 0.0136 mM | Chassagnole, et al. (2002). |
|  | k_pgm,eq_ | 0.196 | Chassagnole, et al. (2002). |
|  | r_max,pgm_ | 0.84 | Chassagnole, et al. (2002). |
| G1PAT | k_g1pat,g1p_ | 3.2 mM | Chassagnole, et al. (2002). |
|  | k_g1pat,atp_ | 4.42 mM | Chassagnole, et al. (2002). |
|  | k_g1pat,fdp_ | 0.119 mM | Chassagnole, et al. (2002). |
|  | n_g1pat,fdp_ | 1.2 | Chassagnole, et al. (2002). |
|  | r_max,g1pat_ | 0.0075 | Chassagnole, et al. (2002). |
| RPPK | k_rppk,rib5p_ | 0.1 mM | Chassagnole, et al. (2002). |
|  | r_max,rppk_ | 0.013 | Chassagnole, et al. (2002). |
| G3PDH | k_g3pdh,dhap_ | 1.0 mM | Chassagnole, et al. (2002). |
|  | r_max,g3pdh_ | 0.012 | Chassagnole, et al. (2002). |
| SER | k_sersynth,3pg_ | 1.0 mM | Chassagnole, et al. (2002). |
|  | r_max,sersynth_ | 0.026 | Chassagnole, et al. (2002). |
| SYN1 | k_synth1,pep_ | 1.0 mM | Chassagnole, et al. (2002). |
|  | r_max,synth1_ | 0.019 | Chassagnole, et al. (2002). |
| SYN2 | k_synth2,pyr_ | 1.0 mM | Chassagnole, et al. (2002). |
|  | r_max,synth2_ | 0.074 | Chassagnole, et al. (2002). |
| DAHPS | k_dahps,pep_ | 0.0053 mM | Chassagnole, et al. (2002). |
|  | k_dahps,e4p_ | 0.035 mM | Chassagnole, et al. (2002). |
|  | n_dahps,e4p_ | 2.6 | Chassagnole, et al. (2002). |
|  | n_dahps,pep_ | 2.2 | Chassagnole, et al. (2002). |
|  | r_max,dahps_ | 0.107 | Chassagnole, et al. (2002). |
| G6PDH | k_g6pdh,nadp_ | 0.0246 mM | Chassagnole, et al. (2002). |
|  | k_g6pdh,nadph,nadpinh_ | 0.01 mM | Chassagnole, et al. (2002). |
|  | k_g6pdh,nadph,g6pinh_ | 6.43 mM | Chassagnole, et al. (2002). |
|  | k_g6pdh,g6p_ | 14.4 mM | Chassagnole, et al. (2002). |
|  | r_max,g6pdh_ | 1.38 | Chassagnole, et al. (2002). |
| PGDH | k_pgdh,6pg_ | 37.5 mM | Chassagnole, et al. (2002). |
|  | k_pgdh,nadp_ | 0.0506 mM | Chassagnole, et al. (2002). |
|  | k_pgdh,atp,inh_ | 208.0 mM | Chassagnole, et al. (2002). |
|  | k_pgdh,nadph,inh_ | 0.0138 mM | Chassagnole, et al. (2002). |
|  | r_max,pgdh_ | 16.23 | Chassagnole, et al. (2002). |
| RU5P | k_ru5p,eq_ | 1.4 | Chassagnole, et al. (2002). |
|  | r_max,ru5p_ | 6.74 | Chassagnole, et al. (2002). |
| R5P1 | k_r5p1,eq_ | 4 | Chassagnole, et al. (2002). |
|  | r_max,r5p1_ | 4.84 | Chassagnole, et al. (2002). |
| TKA | k_tka,eq_ | 1.2 | Chassagnole, et al. (2002). |
|  | r_max,tka_ | 9.47 | Chassagnole, et al. (2002). |
| TA | k_ta,eq_ | 1.05 | Chassagnole, et al. (2002). |
|  | r_max,ta_ | 10.87 | Chassagnole, et al. (2002). |
| TKB | k_tkb,eq_ | 10 | Chassagnole, et al. (2002). |
|  | r_max,tkb_ | 86.56 | Chassagnole, et al. (2002). |
| MUR | r_max,mursynth_ | 0.00044 | Chassagnole, et al. (2002). |
| TRP | r_max,trpsynth_ | 0.001037 | Chassagnole, et al. (2002). |
| MET | r_max,metsynth_ | 0.0022627 | Chassagnole, et al. (2002). |
| PPS | k_m,pyr_ | 8.30E-02 mM | Usuda, et al (2010). |
|  | k_m,atp_ | 2.80E-02 mM | Usuda, et al (2010). |
|  | k_m,pep_ | 3.70E-02 mM | Usuda, et al (2010). |
|  | k_m,amp_ | 1.10E-001 mM | Usuda, et al (2010). |
|  | k_m,pi_ | 38 mM | Usuda, et al (2010). |
|  | P_i_ | 10 mM | Usuda, et al (2010). |
|  | k_pps,eq_ | 875.65 | Usuda, et al (2010). |
|  | r_max,pps_ | 5.71E-003 sec^-1^ | Calculated using Rizzi, et al (1997). |
| FBP | r_max,fbp_ | 3.00E-00 sec^-1^ | Calculated using Rizzi, et al (1997). |
|  | k_m,fdp_ | 1.54E-002 mM | Usuda, et al (2010). |
|  | k_i,amp_ | 2.70E-003 mM | Usuda, et al (2010). |
| Periodic Forcing Function | h | 1 | This Work. |

**Initial Conditions**

| Metabolite | Concentration |  |
| --- | --- | --- |
| C_g6p_ | 3.48 mM | Chassagnole, et al. (2002). |
| C_f6p_ | 0.60 mM | Chassagnole, et al. (2002). |
| C_fdp_ | 0.272 mM | Chassagnole, et al. (2002). |
| C_gap_ | 0.218 mM | Chassagnole, et al. (2002). |
| C_dhap_ | 0.167 mM | Chassagnole, et al. (2002). |
| C_pgp_ | 0.008 mM | Chassagnole, et al. (2002). |
| C_3pg_ | 2.131 mM | Chassagnole, et al. (2002). |
| C_2pg_ | 0.399 mM | Chassagnole, et al. (2002). |
| C_pep_ | 2.67 mM | Chassagnole, et al. (2002). |
| C_pyr_ | 2.67 mM | Chassagnole, et al. (2002). |
| C_6pg_ | 0.8075 mM | Chassagnole, et al. (2002). |
| C_ribu5p_ | 0.111 mM | Chassagnole, et al. (2002). |
| C_rib5p_ | 0.398 mM | Chassagnole, et al. (2002). |
| C_xyl5p_ | 0.138 mM | Chassagnole, et al. (2002). |
| C_sed7p_ | 0.276 mM | Chassagnole, et al. (2002). |
| C_e4p_ | 0.098 mM | Chassagnole, et al. (2002). |
| C_g1p_ | 0.6525 mM | Chassagnole, et al. (2002). |
| C_glc_ex_ | 0.0556 mM | Chassagnole, et al. (2002). |

**Optimization Variables**

| Variable | Initial Value | Constraints |
| --- | --- | --- |
| A | 0.2 | 0.02<A<20 |
| ω | 1.4 | -π<ω<π |
| φ | 0.001 | 0.0005<φ<0.0032 |
